# Rare progerin-expressing preadipocytes and adipocytes contribute to tissue depletion over time

**DOI:** 10.1038/s41598-017-04492-0

**Published:** 2017-06-30

**Authors:** Gwladys Revêchon, Nikenza Viceconte, Tomás McKenna, Agustín Sola Carvajal, Peter Vrtačnik, Peter Stenvinkel, Torbjörn Lundgren, Kjell Hultenby, Irene Franco, Maria Eriksson

**Affiliations:** 1 Department of Biosciences and Nutrition, Karolinska Institutet, 14183 Huddinge, Sweden; 2 Department of Clinical Science, Intervention and Technology, Division of Renal Medicine, Karolinska Institutet, 14186 Stockholm, Sweden; 3 Department of Clinical Science, Intervention and Technology, Division of Transplantation Surgery, Karolinska Institutet, 14186 Stockholm, Sweden; 4 Department of Laboratory Medicine, Karolinska Institutet, 14183 Stockholm, Sweden

## Abstract

Accumulation of progerin is believed to underlie the pathophysiology of Hutchinson-Gilford progeria syndrome, a disease characterized by clinical features suggestive of premature aging, including loss of subcutaneous white adipose tissue (sWAT). Although progerin has been found in cells and tissues from apparently healthy individuals, its significance has been debated given its low expression levels and rare occurrence. Here we demonstrate that sustained progerin expression in a small fraction of preadipocytes and adipocytes of mouse sWAT (between 4.4% and 6.7% of the sWAT cells) results in significant tissue pathology over time, including fibrosis and lipoatrophy. Analysis of sWAT from mice of various ages showed senescence, persistent DNA damage and cell death that preceded macrophage infiltration, and systemic inflammation. Our findings suggest that continuous progerin expression in a small cell fraction of a tissue contributes to aging-associated diseases, the adipose tissue being particularly sensitive.

## Introduction

Chronological aging is a complex process resulting from an accumulation of molecular variations occurring throughout life. One of the major physiological changes that arises with aging is the loss of subcutaneous white adipose tissue (sWAT). White adipose tissue is known to be involved in energy storage, in the form of lipid, but also in immunity, adipokine and inflammatory cytokine production. Different fat depots can be found in both humans and mice, which appear to have distinct functions^1^. Subcutaneous fat, composed of relatively insulin-sensitive small adipocytes, works as an endocrine organ, secreting, in particular, the hormones leptin and resistin. Its role is to store triglycerides and free-fatty acids in order to prevent their ectopic deposition. In the case of lipoatrophy, sWAT’s ability to store energy is impaired, which results in ectopic fat deposition either in visceral depots or in non-adipose sites^2^.

The investigation of premature aging syndromes has had a considerable impact on the understanding of some of the bases of physiological aging. One of these syndromes is the Hutchinson-Gilford Progeria Syndrome (HGPS), commonly known as Progeria, a rare genetic disease characterized by clinical features resembling certain aspects of premature aging. Although several mutations have been reported to cause HGPS, this disease most often results from a *de novo* point mutation in the *LMNA* gene (c.1824C>T; p.G608G*)* ^3–6^. Several of the mutations activate a cryptic splice site that results in the accumulation of a truncated form of prelamin A known as progerin^7^. Progerin accumulates at the inner nuclear membrane causing distortion of the membrane and disrupting nuclear functions^8, 9^. Accumulation of progerin is thought to be responsible for abnormal functional changes associated with HGPS including suppressed Nrf2 antioxidant pathway signalling and impaired adult stem cell function^10, 11^.

Physiological aging has been linked to HGPS on the grounds that they share similar cellular and molecular mechanisms. Moreover, HGPS shares several features with normal aging, one of them being the loss of sWAT. Several studies have revealed the presence of low levels of progerin or rare progerin-expressing cells in normal fibroblasts (between 0.5% and 3%) and arteries (between 0.001% and 1.97%), with amounts sometimes increasing during aging^12–16^. Low tissue levels of progerin can either be attributed to low expression in many cells, or to high expression in a small fraction of cells. However, it is still arguable that low levels of progerin significantly contribute to the reduced tissue function associated with aging^6, 12, 17, 18^. Thus, it remains unclear whether the use of HGPS in studying physiological aging-associated disorders is relevant, and accordingly, the potential involvement of progerin in processes underlying them^19^.

In this study, we used a mouse model with sustained long-term expression of human progerin in a low frequency of cells of the adipose tissue to determine the contribution of progerin to progressive sWAT depletion. Our results provide evidence that adipose tissue is highly sensitive to progerin expression and further emphasize progerin’s possible causal role in certain tissue alterations during aging.

## Results and Discussion

### Progerin is expressed in healthy human sWAT

**Figure 1:**
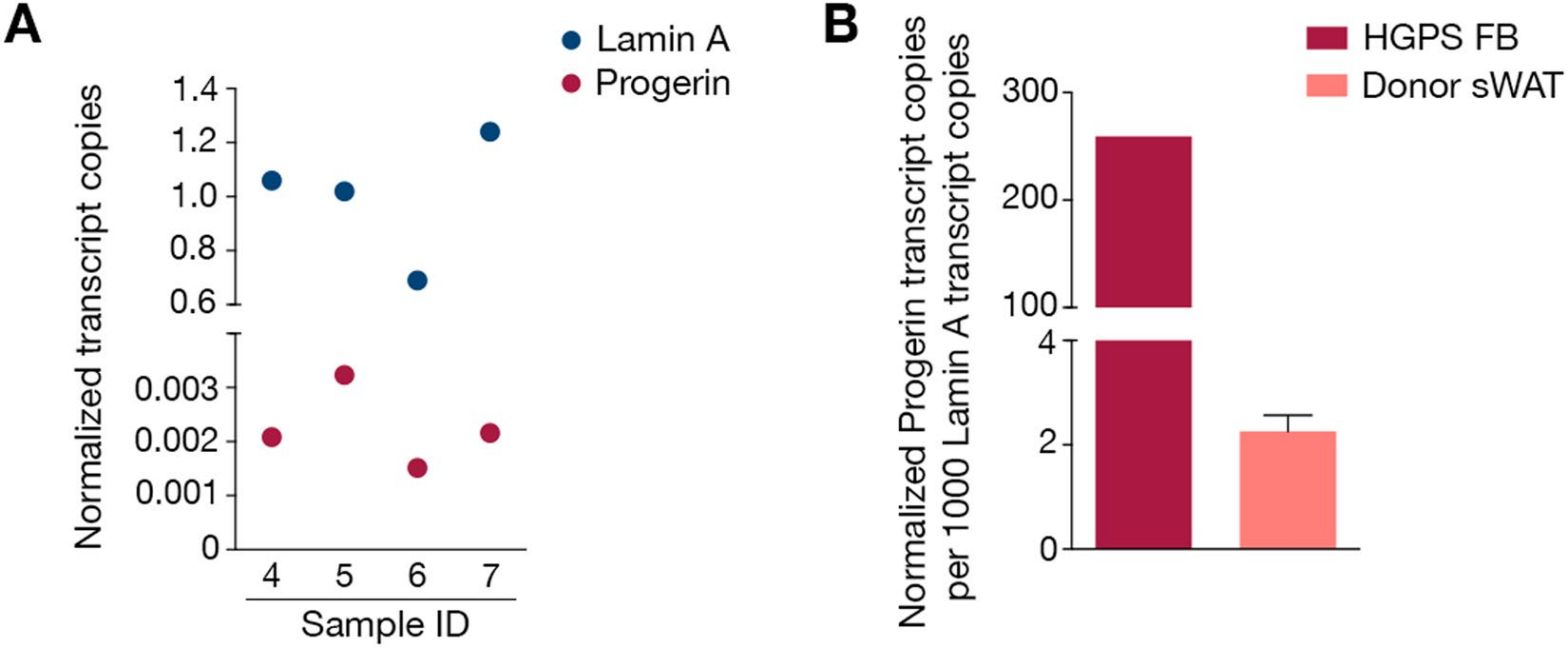
Progerin characterization in human sWAT. (**A**) Graph showing the normalized copy number of progerin and lamin A in healthy human sWAT. (**B**) Graph representing the ratios of progerin to lamin A after normalization to GAPDH as obtained by ddPCR analysis, in HGPS patient fibroblasts (FB) and healthy human sWAT. Data are represented as mean ± SEM.

Expression of progerin has previously been found at low levels in apparently healthy human skin and arteries, with levels increasing with aging^13, 16^. In this study we investigated whether progerin was expressed in human sWAT. Seven biopsies were obtained from healthy individuals (age range: 31–66 years) and assessed for the expression of progerin and lamin A transcripts by digital droplet PCR (ddPCR). The progerin transcript was detected in all seven individuals. In three of the samples, progerin expression was below the limit of quantification and these samples were excluded from further analysis (Supplementary Figure S1). Normalized transcript expression of both progerin and lamin A in different biopsies showed that progerin ranged between 0.0015–0.0032 and lamin A ranged between 0.69–1.24 (Fig. 1A). Ratios of normalized progerin to lamin A transcript copies were calculated. The results revealed that there were 2.26 ± 0.31 copies of progerin per 1000 copies of lamin A in sWAT of healthy individuals (Fig. 1B). To relate this to HGPS, a primary dermal fibroblast sample from a HGPS patient was analyzed for the relative ratios of progerin to lamin A using the same ddPCR assays. The HGPS patient sample had 259.24 copies of progerin per 1000 copies of lamin A (Fig. 1B). Previous studies have shown that levels of progerin vary between different HGPS samples and with passage number^8, 15^. Our results showed that in this HGPS sample, the ratio of progerin to lamin A was 114.77-fold higher when compared to human sWAT (Fig. 1B). However, immunofluorescence analysis of sWAT using a progerin-specific antibody did not reveal any positive cells (n = 5, 31–65 years of age).

### Low frequency of progerin-expressing preadipocytes and adipocytes

Previous gross examination of a HGPS mouse model expressing human progerin and lamin A revealed reduced body weights from 20 weeks of age and complete loss of skin sWAT at 90 weeks^20^. The transgenic expression was driven by the enolase promotor and was mainly expressed in the hippocampus, and to a lesser extent in skin, bone and heart^20^. In the current study, we have used the same strains of mice that we herein refer to as rare progerin expressing mice (RPE mice).

**Figure 2:**
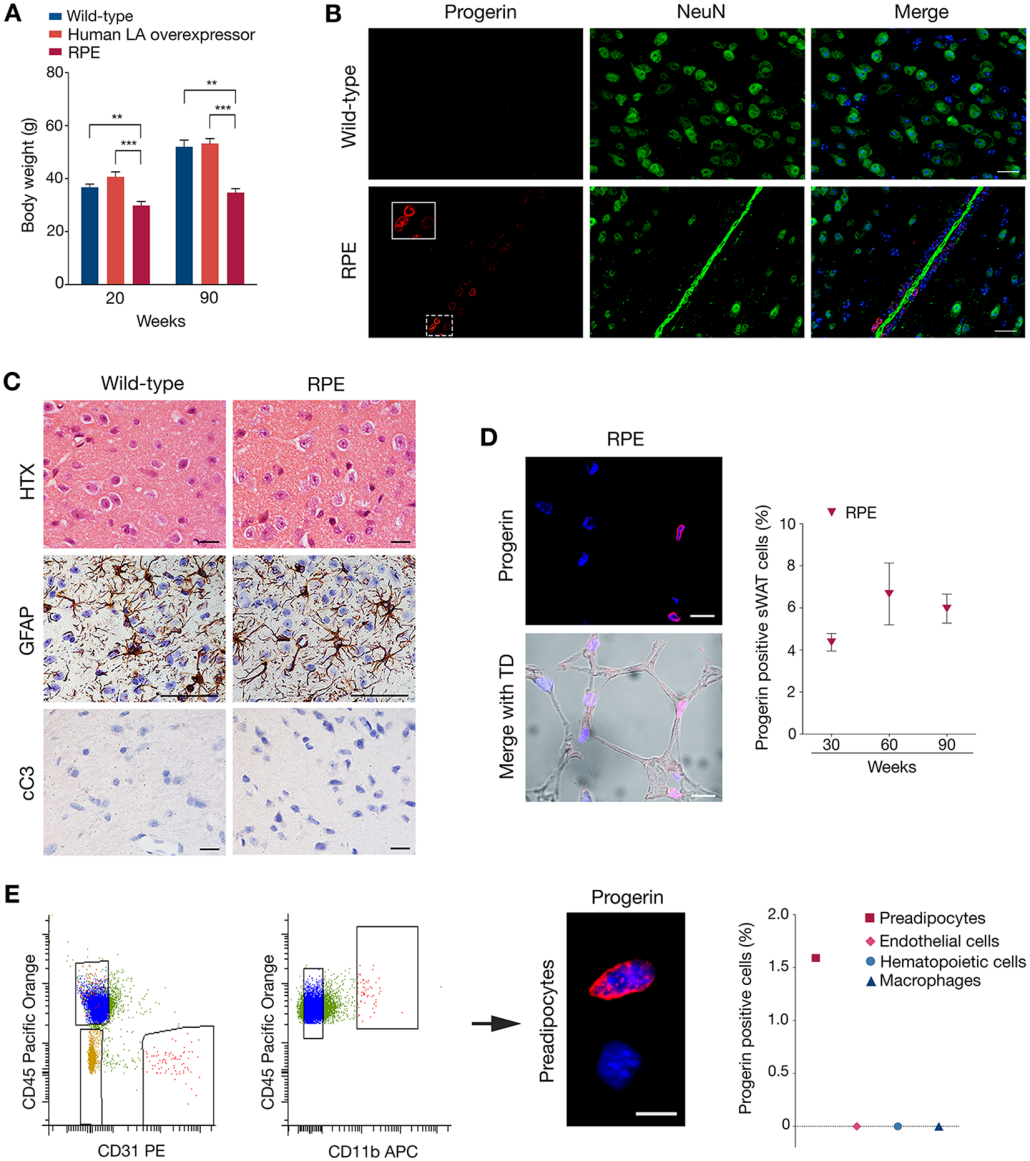
Progerin-expressing sWAT contributes to tissue loss. (**A**) Body weight comparison between wild-type, human lamin A overexpressor and RPE mice. Wild-type and RPE mouse weights were reproduced from Baek *et al*.^19^ (**B**) Immunofluorescent staining of hypothalamic tissues showing progerin (red) together with neuronal cells (green) and cell nuclei (blue) in 60-week wild-type and RPE mice. (**C**) Histological and immunohistochemical stainings on hypothalamic tissue of 60-week control and RPE mice, using either HTX, GFAP or cC3 markers, showing no pathology (upper panel), inactivation of astrocytes (brown) (middle panel) and no apoptosis (bottom panel). (**D**) Representative images and graph of progerin positive cells. Progerin (red) frequency was determined by confocal imaging of RPE sWAT from three age groups, 30, 60 and 90 weeks of age. Transmitted detection (TD) was used to visualize the tissue. (**E**) FACS sorting graphs showing the selected cells for sorting of preadipocytes, endothelial cells and total hematopoietic cells (rectangles, first), which were then sorted as hematopoietic cells and macrophages (rectangles, second). Image showing an example of immunofluorescence against progerin (red) on preadipocytes. Graph (third) illustrating the percentages of progerin positively stained cells per sorted cell types. Scale bars: B = 20 μm, C = 50 μm, D = 10 μm, E = 10 μm. Data are represented as mean ± SEM.

To first determine whether human lamin A overexpression could lead to sWAT loss, we generated a similar mouse model using the same enolase promotor to drive overexpression of wild-type human lamin A^21, 22^. Comparison of body weights from human lamin A overexpressor mice and wild-type mice, at 20 and 90 weeks, revealed no significant difference (Fig. 2A). Skin sections from 120 weeks human lamin A overexpressing mice indicated a hypodermal fat layer similar to the one observed in 90 weeks wild-type mice, indicating no significant effect from the overexpression of human lamin A (Supplementary Figure S2A). In addition, the skin sections from human lamin A overexpressing mice showed a similar frequency and distribution of transgenic human lamin A expression across the skin layers as previously observed in RPE mice^20^, making it an appropriate control (Supplementary Figure S2B). Taken together, our data suggest that the adipose depot was not significantly affected by human lamin A overexpression and that progerin contributed to the observed phenotype in the RPE mice.

Fat metabolism is regulated by the brain through the hypothalamus, which detects circulating fatty acids and blood glucose levels, and responds by controlling feeding behavior and fatty acid metabolism^23, 24^. Thus, we sought to investigate whether progerin expression could affect the functioning of the hypothalamus, consequently leading to fat depletion. Immunofluorescence of progerin in the hypothalami of RPE mice revealed that progerin expression was restricted to a few cells of the vessels, whereas neurons did not show positive staining (Fig. 2B). Moreover, histological analysis showed no evidence of satellitosis (ie. no accumulation of neuroglia cells around neurons or blood vessels) and consequently no pathology (Fig. 2C). Immunostainings indicated that astrocytes remained inactivated and that none of the hypothalamic cells showed evidence of apoptotic state, in either RPE or control mice (Fig. 2C). These findings indicated that the sWAT loss observed in our RPE mice was likely not attributed to hypothalamic defects. For these reasons, we chose to more closely investigate the expression of progerin in sWAT.

To quantify the frequency of progerin-positive cells in sWAT, we performed immunofluorescence using an antibody against human progerin in sections from RPE mice at weeks 30, 60, and 90. Our results showed similar frequency of progerin positive cells in sWAT from the various ages (4.4%, 6.7% and 6% at weeks 30, 60, and 90, respectively), indicating sustained expression with aging (Fig. 2D and Supplementary Figure S2C). Z*mpste24-*deficient mice present accumulation of prelamin A and several features of premature aging^25, 26^. While progeria is caused by the accumulation of a truncated prelamin A protein, with an internal deletion of 50 amino acids, there are obvious similarities, since in both cases prelamin A remains farnesylated and carboxymethylated. A study by De la Rosa and colleagues has demonstrated that prelamin A accumulation in about half of the cells of Zmpste24 mosaic mice does not result in pathology, even up to very old age (>2 years)^27^. In order to determine whether the observed phenotype in the RPE mice might be attributable to a higher frequency of progerin-expressing cells (limited by a possible low sensitivity in our immunofluorescence protocol) progerin on RNA level was analyzed by BaseScope. Similar to the immunofluorescence against progerin, we could only find progerin transcripts in the cytoplasm of a few percent of the cells throughout the sWAT tissue (Supplementary Figure S2D).

In order to identify which cell types were expressing progerin, the main cell populations composing the subcutaneous depot of RPE mice at week 30 were isolated by FACS sorting of the stromal vascular fraction and thereafter stained for progerin. This allowed us to separate myeloid cells/macrophages (CD45^+^ CD11b^+^), non-macrophage hematopoietic cells (CD45^+^ CD11b^−^), endothelial cells (CD45^−^ CD31^+^) and a preadipocyte enriched fraction (CD45^−^CD31^−^)^28, 29^. Progerin positive cells were only found in the preadipocyte enriched fraction (1.59%), while none of the endothelial cells, hematopoietic cells, or macrophages showed expression of progerin (Fig. 2E). These progerin positive cells represented less than 1.17% of the stromal vascular cells. Taken together our results suggested that the low frequency of progerin positive cells in the sWAT of RPE mice consisted of preadipocytes and mature adipocytes.

### Rare progerin-expressing cells result in sWAT depletion with increased age

**Figure 3:**
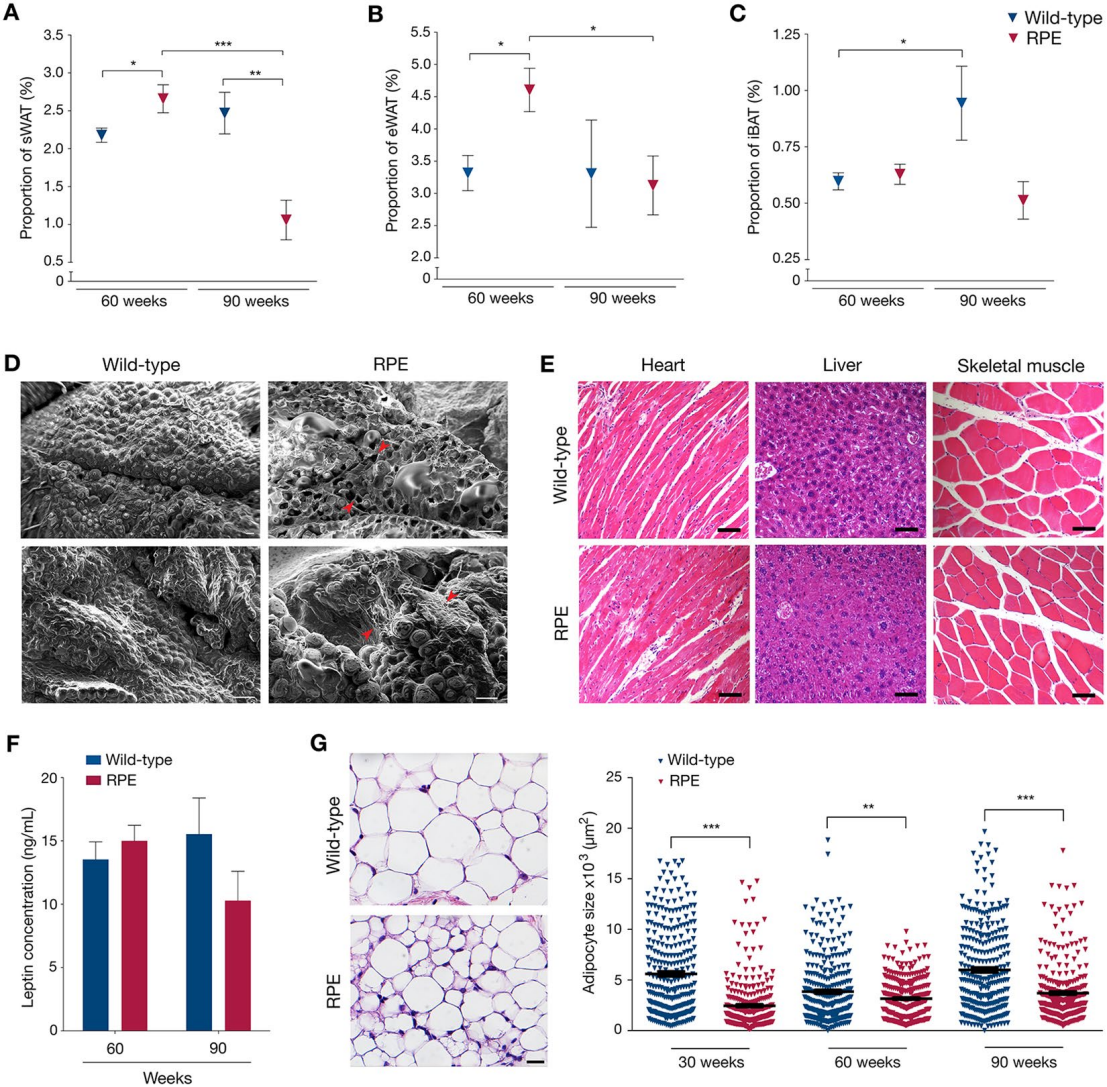
Loss of adipose depots with aging and phenotypic observations. (**A**–**C**) Weights of sWAT (**A**), eWAT (**B**) and iBAT (**C**) depots normalized to body weights of wild-type and RPE animals. (**D**) Representative SEM micrographs of 90-week wild-type and RPE mice, exhibiting adipocyte loss and tissue fibrosis. Red arrows indicate sites of anomaly. (**E**) Histological staining of heart, liver and skeletal muscle showing no accumulation of lipid droplets in either control or RPE mice. (**F**) Graph representing the concentration of circulating leptin at both 60 and 90 weeks of age in either wild-type or RPE mice, as assessed by ELISA. (**G**) Example of HTX staining from 30-week-old wild-type and RPE sWAT, used to estimate average adipocyte surface area (left). Graph representing the adipocyte area of all counted cells in sWAT of 30-, 60- and 90-week old animals (right). Scale bars: D = 100 μm, E = 5000 μm, F = 100 μm. Data are represented as mean ± SEM.

To determine how the different adipose depots were affected by aging in an environment of rare progerin-expressing cells, we isolated and weighed sWAT, epididymal white adipose tissue (eWAT) and interscapular brown adipose tissue (iBAT) from both RPE and control mice. Although RPE mice had greater adipose depots (sWAT and eWAT) than wild-type at 60 weeks, their sWAT was significantly reduced compared to controls at 90 weeks of age (Fig. 3A). This was not evident for the iBAT and eWAT. When comparing 60- to 90-week mice’s fat mass, an appreciable fat loss was observed in sWAT and eWAT of RPE animals over time, sWAT being the most affected of the two (Fig. 3A–C). Scanning electron microscopy (SEM) of sWAT revealed scattered deficits of adipocytes and mild fibrosis in RPE mice compared to control mice at week 90 (Fig. 3D). In agreement with earlier reports, these phenotypic characteristics suggest functional impairment of sWAT with aging^30, 31^. Hence, we sought to investigate signs of metabolic alterations. Dysfunctional and markedly reduced subcutaneous fat, as seen in lipodystrophies, can lead to free fatty acid storage in ectopic sites including the heart, liver and skeletal muscle^32^. Histological stainings of heart, liver and skeletal muscle, were performed to analyze the presence of ectopic fat depots, but displayed no aberrant fat storage in either 90-week RPE or control mice (Fig. 3E). Further analysis of metabolic disturbances via the assessment of circulating leptin concentrations revealed no significant differences when comparing serum levels from RPE and wild-type mice (Fig. 3F), thus indicating that the sWAT function was not severely affected. Histological staining of tissue sections showed that average adipocyte surface area was significantly smaller in RPE mice compared to controls at all time points (Fig. 3G). Taken together, the sWAT depot shows several phenotypic abnormalities and a progressive decline with aging, which may be a result of low-frequency progerin positive cells.

### Increased proliferation, premature senescence and persistent DNA damage in sWAT of RPE mice

**Figure 4:**
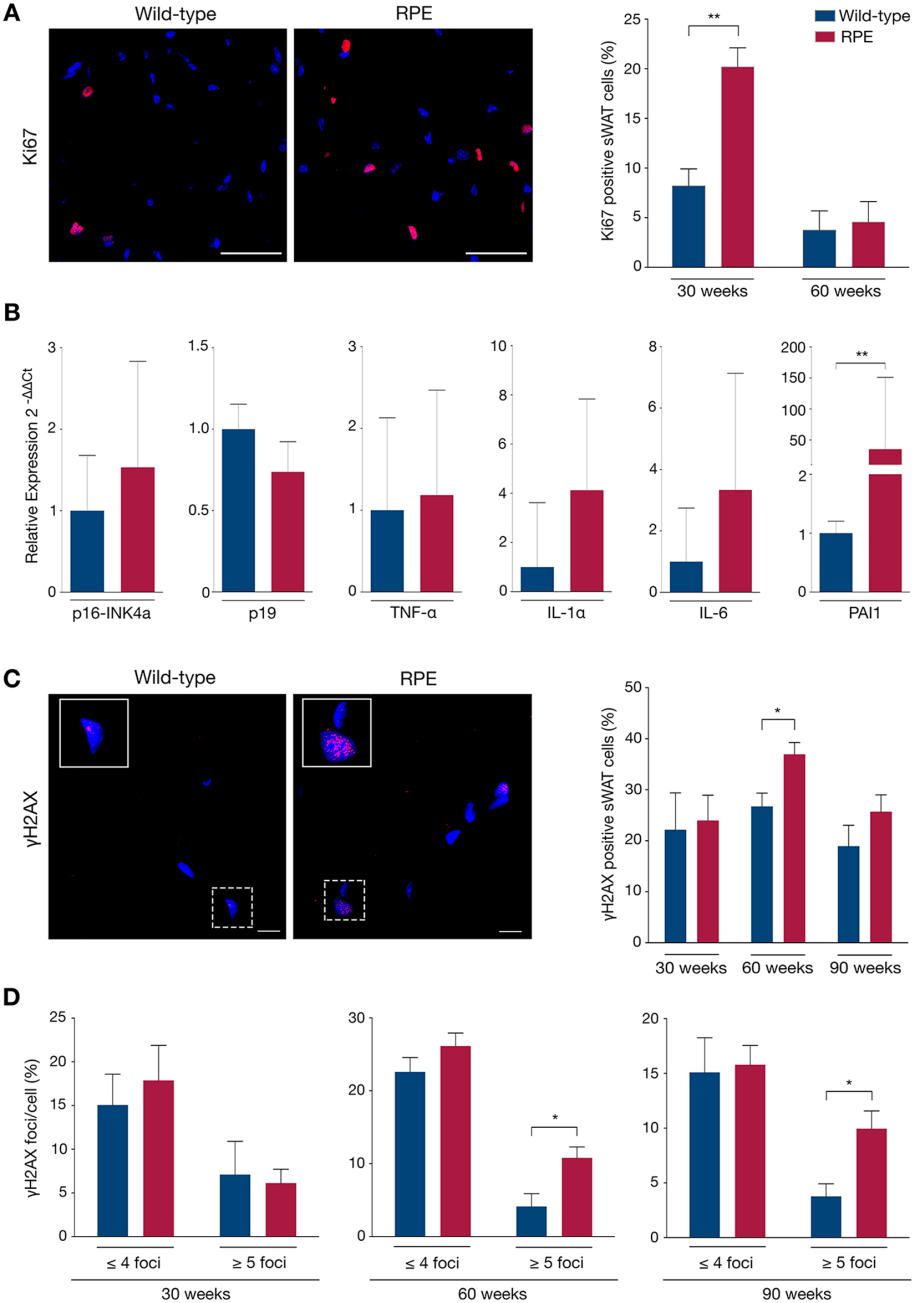
sWAT characterization: Hyperproliferation, senescence and DNA damage. (**A**) Proliferation was determined by immunofluorescent staining of Ki67 (red) in cell nuclei (blue), subsequently analyzed as the percentage of positively stained cells in mutant and control sWAT. (**B**) Senescence was determined via quantitative RT-PCR of specific target genes. (**C**,**D**) γH2AX immunofluorescent staining showing DNA DSBs (red) in cell nuclei (blue) of wild-type and RPE sWAT, first analyzed for the frequency of positive cells (**C**) and then for the frequency of γH2AX foci per cell (**D**). Scale bars: A = 50 μm, C = 10 μm. Data are represented as mean ± SEM.

Changes in the proliferation rate were then investigated. Adipose tissue expansion occurs through two mechanisms: increase in the cell number (hyperplasia) and increase in the cell size (hypertrophy). As previously demonstrated in humans, hyperplasia is negatively correlated with adipocyte hypertrophy^33^. Furthermore, hyperproliferation has previously been observed in cells from HGPS patients and in skin from a progeria mouse model^22, 34^. Analysis of Ki67, a protein observed in actively replicating cells, revealed a 2.5-fold increase in proliferating cells in 30-week RPE sWAT (20.2%) compared to wild-type (8.2%) (p = 0.0095). No difference in proliferating cells was observed at 60 weeks (p = 0.7833) (Fig. 4A). To further investigate whether progerin can induce increased proliferation in sWAT, mouse preadipocytes were transfected with a vector expressing progerin. Analysis of transfected cells using an antibody recognizing human lamin A and progerin showed that 5.15% of the cells expressed progerin. Quantification of proliferating cells showed an increase in proliferation in cells that expressed progerin *vs*. cells transfected with an empty vector (p = 0.0002) (Supplementary Figure S3A).

Increased adipose tissue proliferation can be triggered by several factors, including the impairment of adipocyte metabolism, which is in part dependent on the terminal differentiation process ^33, 35, 36^. The preadipocytes differentiation potential was analyzed *in vitro* following transfection and selection of cells with expression vectors that expressed either human lamin A and progerin, progerin, human lamin A, or an empty vector. No overt differences were visible after ten days of differentiation between the different groups even though 10.2–11.3% of the cells expressed human lamin A and/or progerin (Supplementary Figure S3B). Gene expression analysis of regulators of differentiation (PPAR-γ, C/EBPα, CD36, LPL) revealed a significant down-regulation of C/EBPα (p = 0.0021) and a trend towards a down-regulation of LPL in cells that expressed human lamin A and progerin when compared to empty vector (Supplementary Figure S3C). Similar trends were also observed for cells expressing progerin alone, while there was no difference in the expression levels of PPAR-γ and CD36. Adipocytes that were transfected with human lamin A had similar levels of expression as empty vector-transfected cells for all four genes (Supplementary Figure S3C). This indicated that adipocytes overexpressing both human lamin A and progerin might have a slightly impaired terminal differentiation capacity, which is in agreement with previous reports^37, 38^. Altogether, this suggested that, at early stages, progerin expression in RPE sWAT might alter terminal differentiation causing a defect in lipid metabolism. Thus, RPE sWAT might induce a compensatory mechanism leading to increased proliferation. However this mechanism may not be maintained at later stages. Increased proliferation likely contributes to abnormal cellular development and is known to trigger cell-cycle slowdown and potentially cell-cycle arrest, allowing cells to enter senescence^39^.

Senescence is a hallmark of aging partly responsible for age-dependent decline in tissue function^40^. Senescent cells secrete inflammatory factors that participate in spreading the senescence phenotype to surrounding cells through paracrine activity. This process is referred to as the senescence-associated secretory phenotype (SASP)^41^. It was also demonstrated that aging of adipose tissue results in cellular senescence, which accordingly alters the tissue^42–44^. Thus, we investigated the possibility of cells entering senescence in sWAT. Gene expression analyses of established markers involved in this process (p16^ink4a^, p19, TNF-α, IL-1α, IL6 and PAI1) indicated a shift towards increased senescence in RPE compared to control sWAT already at 30 weeks of age (Fig. 4B). This indicated that senescence might be activated as early as by 30 weeks of age in RPE sWAT, as a result of increased proliferation. Noteworthy, ectopic expression of lamin A was previously found to induce premature senescence in skin fibroblasts^45^, which suggests that together with progerin, lamin A might also play a role in the observed early senescence. Senescence was also evaluated in 90 weeks RPE and control sWAT, by immunostaining against p16. Our results showed an increased frequency of p16 positive cells in RPE sWAT compared to wild-type sWAT (p = 0.028) (Supplementary Figure S3D). Alternatively, senescence can be triggered by DNA damage, especially double-strand breaks (DSBs).

Phosphorylated H2AX (γH2AX) is known to localize at sites of double-strand breaks to form foci^46^. Karakasilioti and colleagues have previously demonstrated in another progeroid mouse model that persistent DNA damage was implicated in adipose tissue depletion through activation of a chronic pro-inflammatory response^47^. Meanwhile increase in persistent DSBs in HGPS patient fibroblasts and cells from HGPS mouse models^12, 48–50^ has already been reported. Analysis of control and RPE sWAT showed increased numbers of γH2AX positive cells at week 60 in RPE (p = 0.0426) (Fig. 4C). In addition both 60- and 90-week RPE subcutaneous depots exhibited a significantly higher number of cells accumulating ≥5 γH2AX foci/nucleus (p = 0.0448 and p = 0.0377 respectively) (Fig. 4D). Taken together, our results indicate that senescence may be more pronounced at later stages, the senescence phenotype being exacerbated by DNA damage and SASP activation, thus triggering the observed sWAT depletion.

### Increased cell death rate and macrophage infiltration in progerin-expressing sWAT

**Figure 5:**
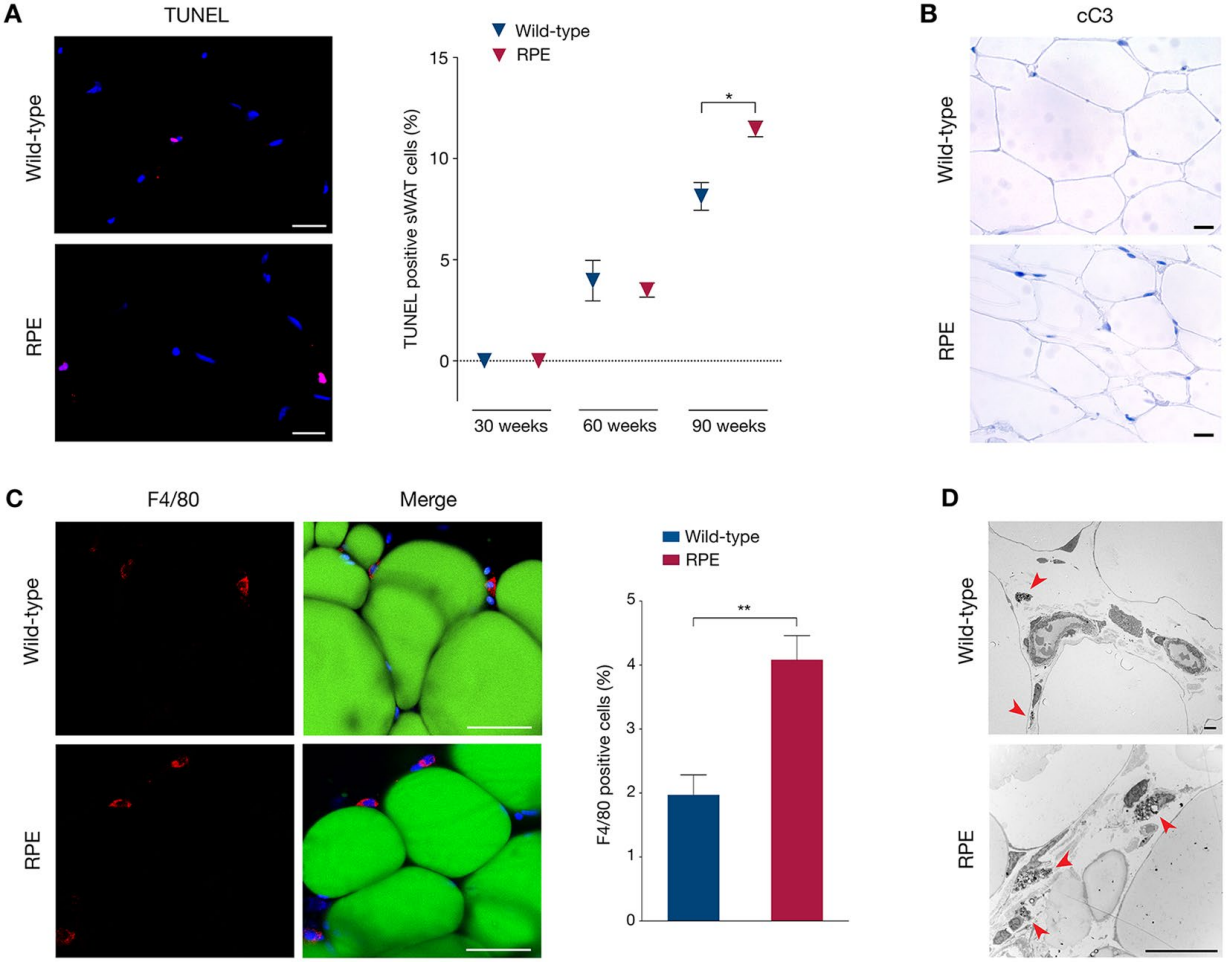
Increased cell death and macrophage infiltration in subcutaneous fat with aging. (**A**) Cell death was assessed using the TUNEL assay on wild-type and RPE sWAT by quantification of positive cells (red). (**B**) Cleaved-caspase 3 (cC3) immunohistochemical staining exhibiting no sign of apoptosis in 90-week wild-type and RPE sWAT. (**C**) Confocal imaging of whole-mount sWAT stained for the macrophage marker F4/80 (red), the adipocyte marker Bodipy® 493/503 (green) and DAPI (blue). Graph representing the frequency of positively stained cells for macrophages in both 60-week RPE and control mice. (**D**) Representative TEM micrographs of 90-week sWAT from wild-type and RPE animals showing infiltrating macrophages (red arrows) in progerin-expressing tissues. Scale bars: A = 20 μm, B = 100 μm, C = 50 μm, D = 20 μm. Data are represented as mean ± SEM.

Many factors can trigger the apoptotic determination of a cell, including DNA damage^51, 52^. It has also been hypothesized that with hyperproliferation, nuclei of cells resulting from each round of cell division might develop abnormally, which could lead to apoptosis^34^. Since RPE sWAT cells have increased numbers of γH2AX foci evident only from week 60, and higher rates of proliferation at 30 weeks of age, we investigated signs of apoptosis. Increased cell death, as detected by the TUNEL assay, was observed in the 90-week RPE mice compared to controls (p = 0.0134) (Fig. 5A and Supplementary Figure S3E). Primary immunostaining for cleaved-caspase 3 did not identify apoptotic cells in 90-week RPE and control sWAT (Fig. 5B). These results indicated that cell death occurs via a non-caspase 3-mediated pathway.

Dead cells are cleared by macrophages, which infiltrate damaged tissues and boost tissue inflammation. In addition, it has previously been demonstrated that macrophage infiltration of the adipose tissue is promoted by DNA damage^47, 53^. Infiltrating macrophages have also been found in all adipose depots of an established lipodystrophic mouse model^54^. Therefore, we investigated the possibility that macrophages play a role in the fat depletion occurring in these RPE mice. Analysis by whole-mount fluorescence microscopy using the macrophage marker F4/80 on 60-week RPE and control sWAT, revealed a 2-fold increase in F4/80 positively stained macrophages in RPE sWAT compared to wild-type (p = 0.0049)(Fig. 5C). An increased number of macrophages was also observed by transmitted electron microscopy (TEM) analysis of sWAT from 90-week RPE mice compared to controls (Fig. 5D). Our results suggested that increased DNA damage at 60 weeks, together with increased cell death at 90 weeks, triggered macrophage accumulation in RPE mouse sWAT.

### Rare progerin expression in sWAT results in an activated systemic inflammatory response

**Figure 6:**
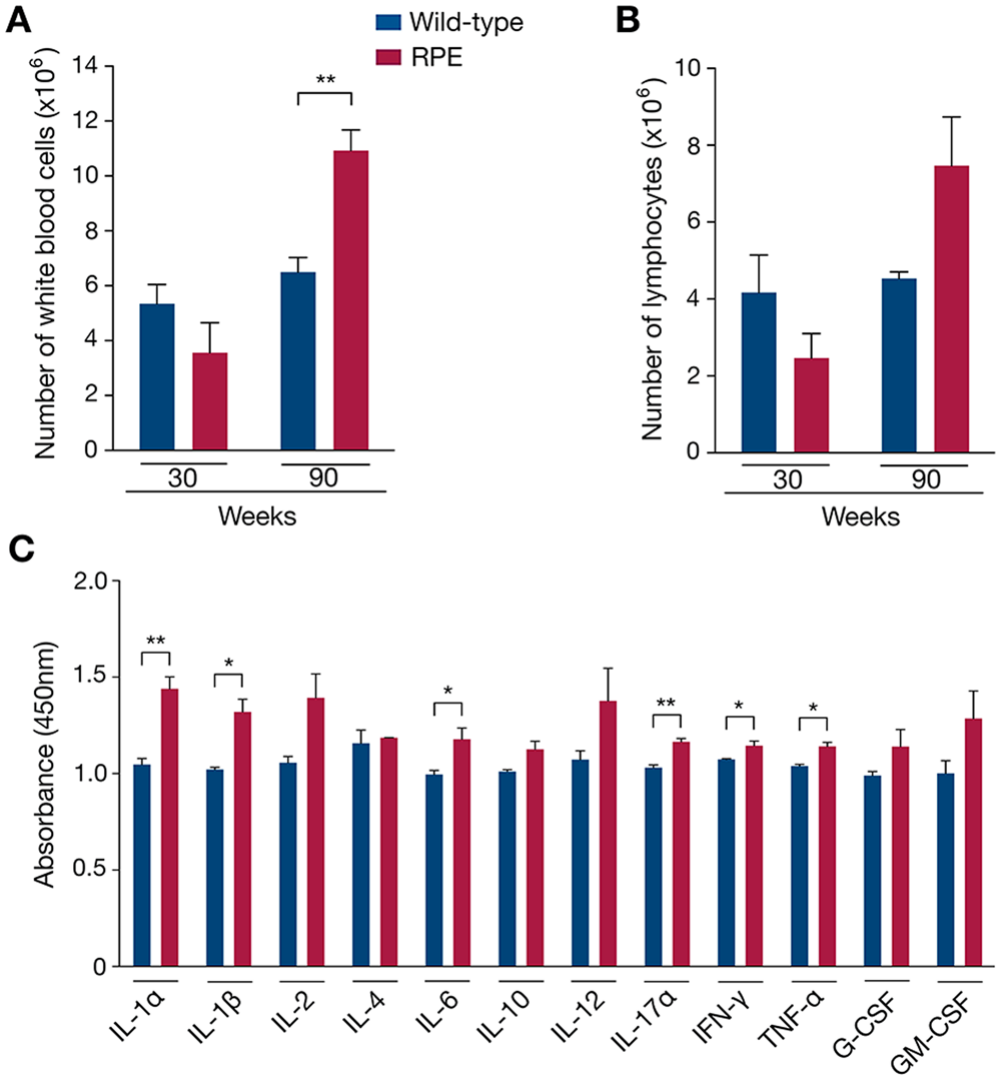
Systemic inflammation activation in response to sWAT depletion in RPE mice. (**A**–**C**) Systemic inflammation was evaluated first by whole-blood analysis, giving the number of total white blood cells (**A**) and lymphocytes (**B**), then by ELISA giving the levels of circulating pro-inflammatory cytokines (**C**), in RPE and wild-type mice.

Adipose tissue pathology has been associated with systemic inflammation, characterized by increased levels of circulating pro-inflammatory cytokines, such as interleukin-6 (IL-6) and tumor necrosis factor alpha (TNF-α)^54^. In addition, it is thought that the adipose tissue produces pro-inflammatory cytokines and adipokines, which proceed in a paracrine and systemic manner, and are capable of activating both the adaptive and innate immune responses^55, 56^. Therefore, we investigated the presence of systemic inflammation. While the number of circulating leukocytes was similar in 30-week wild-type and RPE mice (Fig. 6A), a significantly elevated number of white blood cells was detected in 90-week RPE mice (p = 0.0083), with an increase in circulating lymphocytes (Fig. 6B), suggesting systemic immune activation. To further explore this possibility, we performed ELISA to measure serum cytokines at week 90 in RPE and wild-type controls. The pro-inflammatory cytokines IL-1α, IL-1β, IL-6, IL-17α, interferon gamma (IFNγ) and TNF-α were significantly higher in RPE compared to wild-type controls (p < 0.05 for IL-1β, IL6, IFNγ and TNF-α; p < 0.005 for IL-1α and IL-17α) (Fig. 6C). These results suggest that the immune response is stimulated in RPE mice that have had sustained expression of progerin in sWAT up to week 90.

## Conclusion

**Figure 7:**
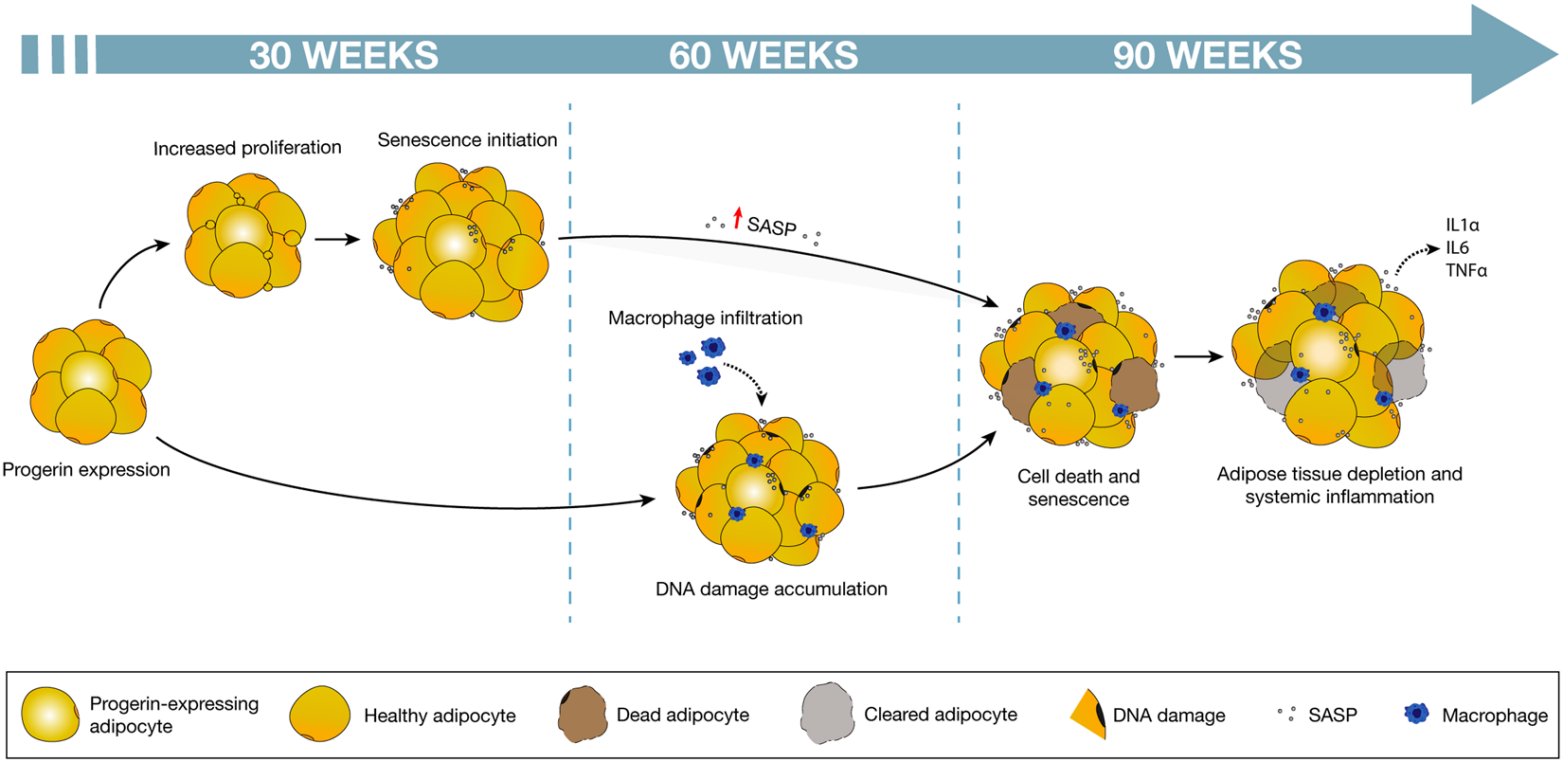
Proposed mechanism of adipose tissue depletion caused by low levels of progerin expression. Low frequency of progerin-expressing cells resulted in increased proliferation and initiation of senescence in 30 weeks RPE sWAT. At 60 weeks of age, the sWAT cells had accumulated DNA DSBs and macrophages had been infiltrating the tissue. At the last time point analyzed (from 90 weeks), cell death and senescence were increased, leading to tissue depletion and induction of systemic inflammation.

In summary, we have demonstrated that rare progerin-expressing preadipocytes and adipocytes trigger subcutaneous adipose tissue depletion over time. However, the frequency of progerin positive cells in the sWAT of our mouse model was higher than what was observed in healthy human sWAT, in which progerin could not be detected on protein level. In 2010, Tchkonia and colleagues suggested a hypothetical model whereby aging of adipose tissue results in cellular senescence and consequent tissue pathology^55^. Our results provide evidence that a similar mechanism is to be found in subcutaneous fat, with progerin accumulation during aging triggering a cascade of events contributing to progressive tissue depletion. We propose that with chronic exposure to low numbers of progerin expressing cells, sWAT pathology begins, initially with hyperproliferation. Hyperproliferation in turn contributes to abnormal cellular development and subsequent senescence. As paracrine activity is high in adipose tissue, senescence spreads to surrounding cells through activation of the SASP. Simultaneously, aging sWAT accumulates DNA DSBs, which upon reaching a certain threshold lead to an increase in cell death, encouraging macrophage infiltration, as well as exacerbating the senescence phenotype. This pro-inflammatory environment of the adipose tissue ultimately activates the immune system machinery resulting in systemic inflammation (Fig. 7).

## Experimental Procedures

### Transgenic mice

Animal studies were approved by the Stockholm South Ethical review board, Dnr. S101-12, 35–15. All procedures were performed in accordance with the institutional guidelines and regulations. Mice were housed in a pathogen-free animal facility at the Karolinska Institutet, Huddinge, Sweden, and maintained in a 12-hour light/dark cycle, at 20–22 °C temperature and 50–65% air humidity. Heterozygous animals carrying the tissue-specific promoter-regulated transactivator NSE-tTA (on CD-1 genetic background)^21^ were crossed with either heterozygous tetop-LA^G608G^ animals (on FVB/NCrl genetic background) carrying the human lamin A minigene containing the most common HGPS mutation, *LMNA* c.1824C>T; p.G608G (tetop-LA^G608G^; F1-line VF1-07) or tetop-LA^wt^ animals (on FVB/NCrl genetic background) carrying the human lamin A minigene (tetop-LA^wt^; F1-line SF1-04)^22^. PCR genotyping was performed according to previously described procedures^20^. Offsprings that were positive for both transgenes, tetop-LA^G608G+^; NSE-tTA^+^, were referred to as rare progerin expressing mice (RPE mice); tetop-LA^wt+^; NSE-tTA^+^, were referred to as human lamin A overexpressor mice. Non-transgenic offspring, tetop-LA^G608G−^; NSE-tTA^−^, and tetop-LA^G608G+^; NSE-tTA^−^ were referred to as wild-type and were used as controls, exposed to the same conditions as the RPE mice. Body weights were measured at 20 weeks (n = 29 for wild-type, n = 11 for human lamin A overexpressor mice and n = 13 for RPE) and 90 weeks (n = 4 for wild-type, n = 9 for human lamin A overexpressor mice and n = 4 for RPE). Mice were normalized between the different groups in regard to age and gender. Experiments were conducted on three age groups: 30-week, 60-week and 90-week. Mice were sacrificed by cervical dislocation after administering an excess of isofluran.

### Human tissue and cell samples

The study was approved by the Stockholm Regional Ethical review board, Dnr. 2015/1115-31. All procedures were performed in accordance with the institutional guidelines and regulations. Human subcutaneous adipose tissue samples were obtained from the Karolinska University Hospital, Department of Transplantation Surgery. Informed consent was obtained from all subjects. The biopsies originated from seven healthy kidney donors undergoing surgery. Information such as age and gender are presented in the Supplementary Methods. Part of the biopsies were fixed in 4% paraformaldehyde (for later immunostaining) and remaining tissues were stored at −80 °C (for later gene expression analysis). Primary dermal fibroblasts from the HGPS patient included in this study (AG06917) were obtained from the Coriell Cell Repositories (Camden, USA). Cells had previously been grown and RNA isolated in our laboratory as previously described^14^.

### Gene expression analysis

Gene expression analysis was performed on cDNA from 30-week-old RPE and control mouse sWAT (n = 3/group) using quantitative RT-PCR, and human sWAT (n = 7) using droplet digital PCR (ddPCR). Primer sequences and qPCR conditions are provided in the Supplementary Methods. Relative qPCR analysis was performed in accordance with previously described procedures^57, 58^. ddPCR was carried out as previously described^59^.

### Adipose depots analysis

Subcutaneous white adipose tissue, epididymal white adipose tissue and interscapular brown adipose tissue from 60- and 90-week-old RPE (males, n = 11) and control mice (males, n = 12) were isolated and weighed. Fat depot weights were normalized to mouse body weights.

### Tissue sections histology and immunostaining

For histology, isolated tissues were fixed in 4% paraformaldehyde, embedded in paraffin and cut into 4 μm sections (n ≥ 3/group). Histological analysis was performed by staining with haematoxylin and eosin (HTX), according to standard procedures. Adipocyte area was measured using the Adiposoft software. A total of one hundred cells per sample were analyzed. For immunostaining, tissue sections were deparaffinized and subjected to heat-induced epitope retrieval. Specimens were blocked and primary antibodies were applied to sections followed by overnight incubation. Sections were incubated with secondary antibodies. For immunohistochemistry, the label antibody was added (ABC Elite, Vector Laboratories) and enzymatic activity observed. Tissue sections were counterstained either with Mayers haemotoxylin (Histolab) or DAPI (ThermoFisher Scientific). Detailed protocols, antibodies, vendors and concentrations used in the present study are presented in the Supplementary Methods.

### Whole-mount immunostaining

Fresh sWAT from 60-week-old RPE and control animals (males, n = 3/group) were thinly sliced (<2mm). Cells were permeabilized and non-specific bindings were blocked. Samples were first incubated with the primary antibodies, then with the appropriate secondary antibodies, Bodipy® 493/503 (ThermoFisher Scientific) and DAPI. The detailed protocol is presented in the Supplementary Methods.

### Scanning Electron Microscopy and Transmission Electron Microscopy

sWAT from 90-week RPE and wild-type mice (n = 3/group) were dissected and small pieces were fixed. For SEM, specimens were dehydrated then mounted on an aluminium stub and coated with Platinum (Bal-Tec SCD 005). For TEM, specimens were dehydrated and embedded. Ultra-thin sections were cut and contrasted with uranyl acetate followed by lead citrate. Further information can be found in the Supplementary Methods.

### TUNEL assay

Cell death was determined using the *In Situ* Cell Death Detection Kit, TMR red (Roche) in accordance with the manufacturer’s protocol, comparing RPE and wild-type mice sWAT (n = 3/group/age). Nuclei were counterstained with DRAQ5™ (1:1000, ThermoFisher Scientific).

### ELISArray

Determination of leptin concentrations was carried out on 60- and 90-week-old RPE and control mouse serum (n ≥ 3/group) using the Mouse Leptin ELISA Kit (Millipore). Quantification of inflammatory cytokines was performed on 90-week-old RPE and control animal serum (males, n = 3/group) using the Mouse Inflammatory Cytokines Multi-Analyte ELISArray kit (Qiagen). For both assays, absorbances were measured following the manufacturer’s protocol, using the Infinite 200Pro plate reader (Tecan, Switzerland).

### Fluorescence-activated cell sorting analysis (FACS)

Stromal vascular cells (SVCs) extraction for FACS analysis was done on 30-week RPE animals (n = 3) as previously described^28^. Briefly, SVCs (composed of preadipocytes, endothelial cells, T cells, B cells and macrophages) were collected from the pellet obtained after centrifugation of collagenase-digested sWAT, while floating mature adipocytes were discarded. After clearing of red blood cells, SVCs were stained for cell surface markers against endothelial cells (CD31), hematopoietic cells (CD45) and macrophages (CD11b). Detailed protocol and information regarding the antibodies used are presented in the Supplementary Methods. Samples were analyzed using a BD FACSAria™ Mu cell sorter (BD Biosciences, USA). Sorted cells were cytospun using the Cytospin™ 4 Cytocentrifuge (Thermo Scientific, USA) and subsequently immunostained using an antibody against Progerin (1:150). Given the low amount of endothelial cells and macrophages obtained, data from the 3 mice were pooled for each cell type to reach a minimum of 100 cells. Between 120 and 4095 cells were counted for each cell type.

### Whole-blood analysis

Blood from 30- and 90-week-old RPE and wild-type mice (n = 3/group) was collected from the vein tail into lithium-heparin Microvette® 200 (Sarstedt). Samples were processed with the Sysmex XP-300™ Automated Hematology Analyzer (Sysmex Corporation, Japan) or treated with Red Blood Cell lysis solution (Qiagen). Thereafter, white blood cells were manually counted using a haemocytometer.

### Statistical analysis

All p-values were calculated using 2-tailed distribution, 2-sample unequal variance Student’s *t*-test with significance defined as p < 0.05 (*p < 0.05, **p < 0.005, ***p < 0.0005). Results are presented as mean ± standard error of the mean (SEM). Correlations were performed using Pearson correlation coefficients with a 2-tailed 95% confidence interval. All calculations were performed using GraphPad Prism software.

## Electronic supplementary material

Supplementary Figures and Methods
