# Supplementary material for: Rare progerin-expressing preadipocytes and adipocytes contribute to tissue depletion over time
Source: Sci Rep. 2017 Jun 30;7:4405. doi: 10.1038/s41598-017-04492-0 (PMC5493617; doi:10.1038/s41598-017-04492-0)
Supplement: Supplementary file 1 — Supplementary Figures and Methods [file 41598_2017_4492_MOESM1_ESM.pdf]

Rare progerin-expressing preadipocytes and adipocytes contribute to tissue depletion over time.

Gwladys Revêchon, Nikenza Viceconte, Tomás McKenna, Agustín Sola Carvajal, Peter Vrtačnik, Peter Stenvinkel, Torbjörn Lundgren, Kjell Hultenby, Irene Franco, Maria Eriksson

SUPPLEMENTARY INFORMATION

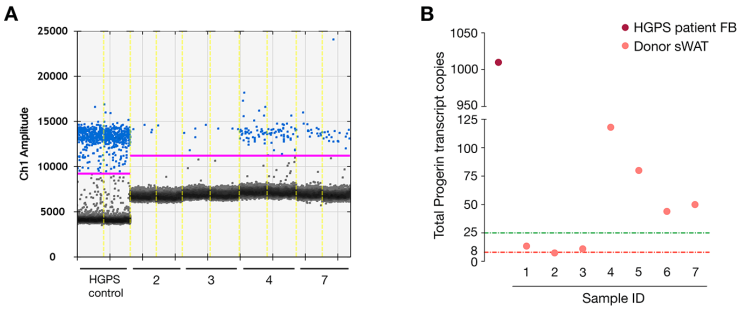

Supplementary Figure S1. Progerin expression in human sWAT analysis.
A. Graph representing the number of droplets obtained by ddPCR on human sWAT assessed for progerin expression. **B.** Graph showing the samples detected and used for quantification of progerin, as determined by the LOD (red axis) and LOQ (green axis).

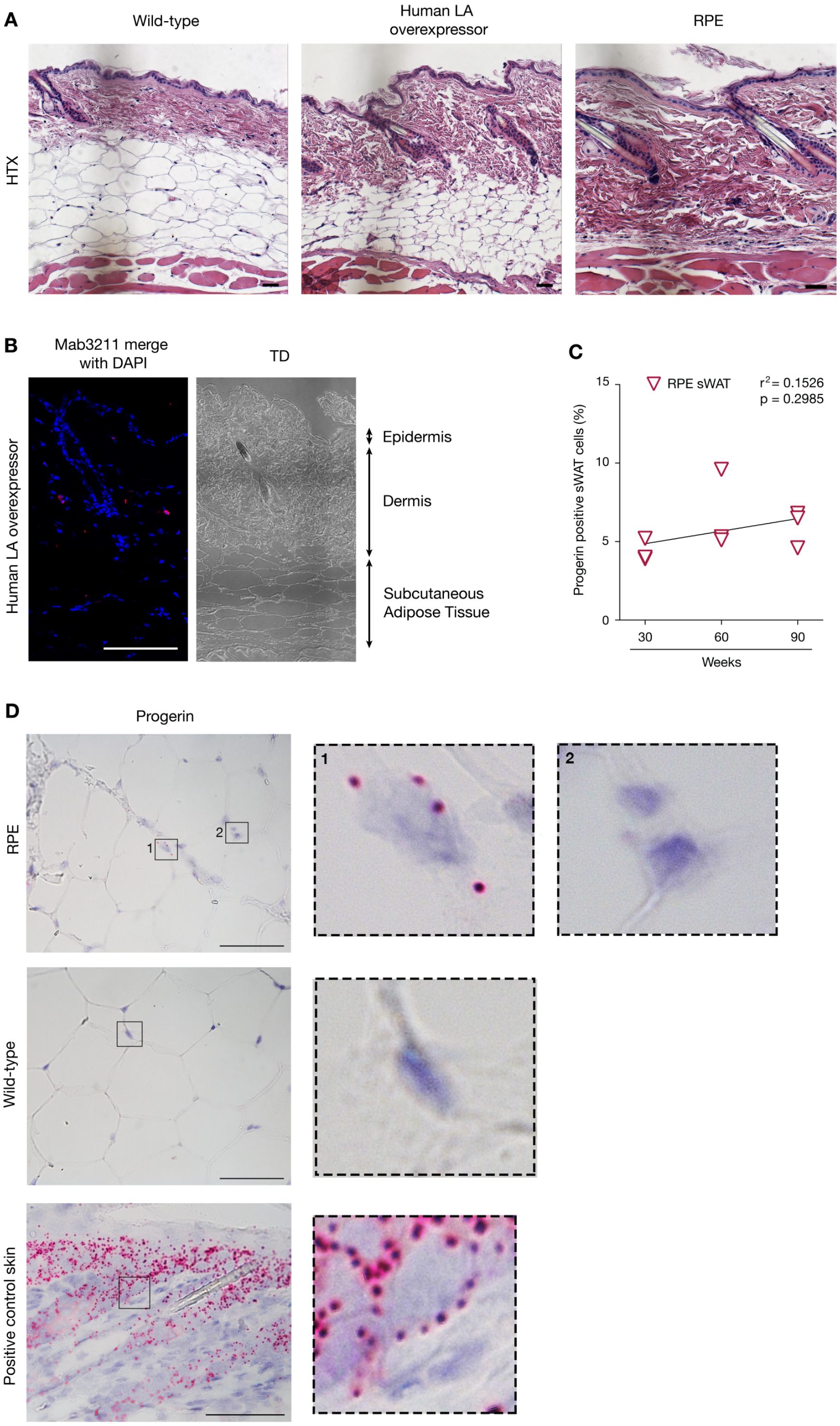

Supplementary Figure S2. Involvement of lamin A and progerin in adipose tissue depletion.
A. Haematoxylin staining of skin sections from 90 weeks wild-type and RPE mice, as well as from 120 weeks human lamin A overexpressor mice. **B.** Immunofluorescence showing human lamin A overexpression in the dermal and hypodermal skin layer (Mab3211 positive-red). Transmitted detection was used to visualize the different skin layers. **C.** Graph indicating no aging-correlated accumulation of progerin-expressing cells in RPE mouse sWAT. **D.** BaseScope showing progerin transcripts in 30 weeks RPE sWAT, absence of progerin in 30 weeks wild-type sWAT and progerin overexpression in the skin from a mouse with Keratin-5 controlled overexpression of progerin overexpressing progerin used as a positive control. Scale bars: A = 90µm, B = 100µm, D = 70µm.

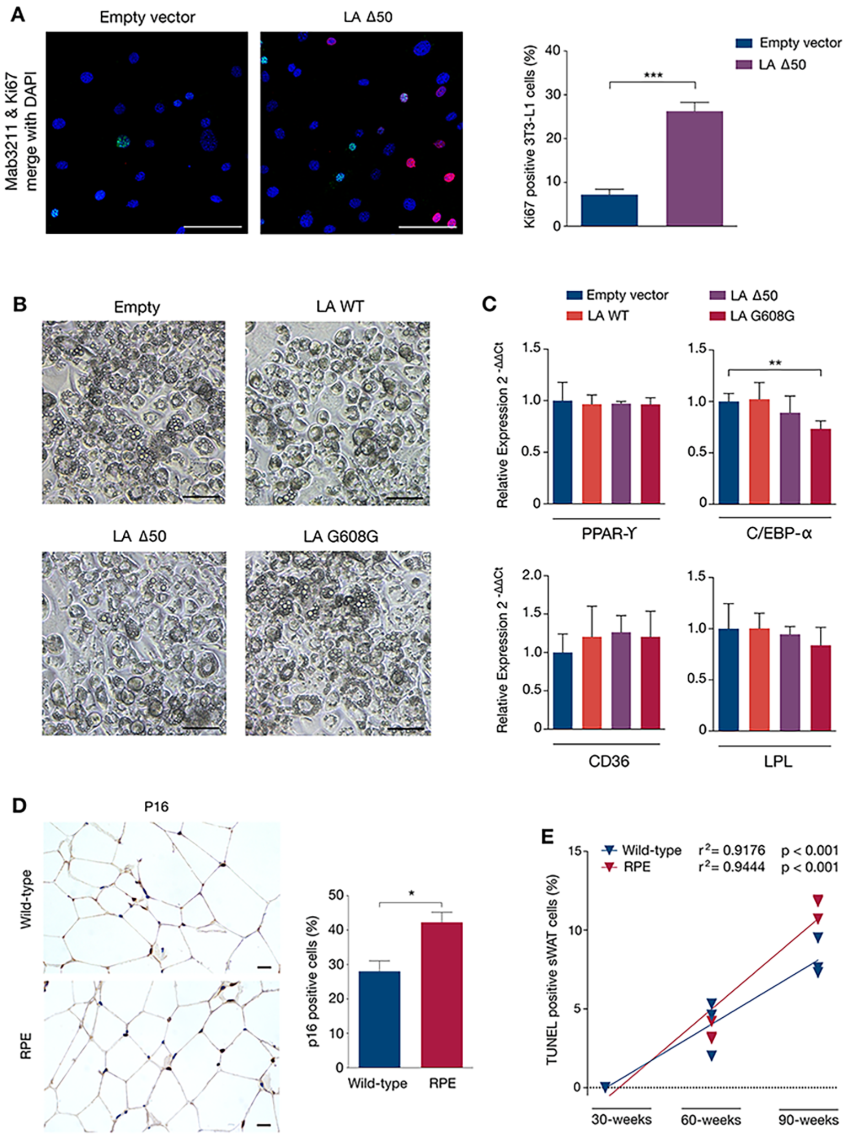

Supplementary Figure S3. sWAT damages induced by low expression of progerin and lamin A. **A.** Immunofluorescent staining of 3T3-L1 preadipocytes transfected to express progerin (LA Δ50) or with an empty vector, showing proliferating cells (Ki67 positive-green) and progerin-expressing cells (Mab3211 positive-red). Data were analyzed for the frequency of Ki67 positive cells. **B.** Representative pictures of differentiated preadipocytes, transfected beforehand with an empty vector or to express progerin (LA Δ50), human lamin A (LA WT) or both human lamin A and progerin (LA G608G). **C.** Graphs representing gene expression analysis of PPAR-γ, C/EBPα, CD36 and LPL in differentiated preadipocytes transfected with an empty vector, LA Δ50, LA WT or LA G608G. **D.** p16 immunostaining showing senescent cells (brown) in sWAT from 90 weeks RPE and wild-type mice, and analyzed for the frequency of positive cells. **E.** Graph showing accumulation of cell death in both wild-type and RPE mice, in correlation with aging. Scale bars: A = 100µm, B = 170µm. Data (A. C. & D.) are represented as mean ± SEM.

SUPPLEMENTARY METHODS

Donor's informations

Donor ID	Age (Years)	Gender
1	31	Male
2	52	Female
3	52	Female
4	57	Female
5	57	Male
6	65	Female
7	66	Female

Cell culture, transfection and adipocyte differentiation.
3T3-L1 preadipocytes were cultured in DMEM (Gibco) supplemented with 10% NCBS and 1% penicillin/streptomycin (Gibco) at 37 °C with 5% CO_2. For transfection, cells were plated at 70% confluence in a 6-well plate and transfected using Lipo3000 (Invitrogen) according to the manufacturer's instructions. A pcDNA3.1 vector carrying the LMNA minigene (reference) (LA WT), LMNA minigene with G608G mutation (LA G608G), cDNA for progerin production only (LA Δ50) or empty vector was used for transfection. After 48h the cells were transfered to a T25 flask and selected using 1mg/ml geneticin (Gibco). Media was changed daily for 2 weeks and the selected cells were used for differentiation as follows: cells were plated in a 6-well plate. When the cells reached 90% confluence, differentiation was initiated by changing media to DMEM 10% FBS + 5 μl/ml insulin, 1 μM dexamethasone and 0.5 mM IBMX (Sigma). Three days after, media was changed to DMEM 10% FBS + 5 μl/ml insulin to maintain the differentiation. Cells were collected for RNA extraction or fixed in 4% PFA for immunofluorescence at day 10 after differentiation induction.

Gene expression analysis
RNA was extracted from 30-week-old RPE and control mouse sWAT (n = 3/group) and human sWAT (n = 7) using TriZol® Reagent (Invitrogen) according to standard procedures. RNA was treated with DNAse I (RQ1 RNase-free DNAse, Promega) according to the manufacturer's recommendations, and cleaned-up using RNeasy mini-columns (Qiagen). cDNA synthesis was performed using random hexamers and SuperScript Reverse Transcriptase (Invitrogen).

Quantitative RT-PCR was performed on mouse cDNA. Each experiment (p19, TNFα, IL1α, IL6, PAI1, PPAR-γ, C/EBP-α, CD36, LPL and β-actin) was performed on cDNA or no template control, using 2x Power SYBR® Green and 10μM of the respective forward and reverse primers. qPCR was performed in a total reaction volume of 20μL containing 5μL of diluted cDNA in MicroAmp 96-well plates. Plates were loaded into an ABI7500 fast system sequencing detection instrument (Applied Biosystems). The following cycling conditions were used: 2 min 50°C, 10 min 95°C, 40 cycles of 15 sec 95°C and 40 sec 60°C. All reactions were run in triplicate. Tbp and p16 qPCRs were run with Taqman qPCR reagents and primers, and performed as previously described[1]. Briefly, 1μL cDNA was mixed with 10μL Taqman Universal Mastermix (Invitrogen) and 1μL Taqman primers in 8μL of water. The following cycling conditions were used: 2 min 50°C, 10 min 95°C, 40 cycles of 15 sec 95°C and 1 min 60°C. All reactions were run in triplicate. Relative qPCR analysis was performed in accordance with previously described procedures[2].

Droplet digital PCR (ddPCR) was carried out on human sWAT cDNA as previously described[3]. Briefly, 20μL PCRs were performed in Twin Tec semiskirted 96-well plates (Eppendorf) using QX200 ddPCR EvaGreen Supermix (BioRad) according to the manufacturer's recommendations. The following cycling conditions were used: 5 min 95°C, 40 cycles of 30 sec 95°C and 1 min 60/63°C (for both GAPDH and Lamin A, and for progerin respectively), 5 min 4°C, 5min 90°C. Analysis was performed using the QX200 droplet digital PCR system (BioRad, Sweden). Limit of quantification (LOQ) was defined as the lowest concentration measured with a coefficient of variation of 20% or less. Limit of detection (LOD) was established by serial dilutions of a cDNA control sample. The LOQ of our assay was determined to be 25 copies whereas limit of detection (LOD) was 8 copies. Samples below LOQ were not included in the quantification.

Primers list

Primer name		Sequence	Probe
p19	Forward<br>Reverse	5′-GCCGCACCGGAATCCT-3′<br>5′-TTGAGCAGAAGAGCTGCTACGT-3′	
TNF-α	Forward<br>Reverse	5'-AAGGGAGAGTGGTCAGGTTGCC-3'<br>5'-CCTCAGGGAAGAGTCTGGAAAGG-3'	
IL-1α	Forward<br>Reverse	5'-CAGGATGTGGACAAACAC-3'<br>5'-GCTCACGAACAGTTGTGAATCTG-3'	
IL-6	Forward<br>Reverse	5'-ATCCAG TGCCTTCTTGGGACTGA-3'<br>5'-TAAGCCTCCGACTTGTGAAGTGGT-3'	
PAI1	Forward<br>Reverse	5′-TCAGAGCAACAAGTTCAACTACACTGAG-3<br>5'-CCCACTGTCAAGGCTCCATCACTTGGCCCA-3′	
β-actin	Forward<br>Reverse	5'-CCTAGGCACCAGGGTGTGAT-3'<br>5'-CCATGTCGTCCCAGTTGGTAA-3'	
p16	Forward<br>Reverse	5'-CCCAACGCCCCGAACT-3'<br>5'-GCAGAAGAGCTGCTACGTGAA-3'	5'-TTCGGTCGTACCCCGATTCAGGTG-3'
Tbp	Forward<br>Reverse	Assay from Life Technologies Mm00446973_m1	
Progerin	Forward<br>Reverse	5'-ACTGCAGCAGCTCGGGG-3'<br>5'-TCTGGGGGCTCTGGGC-3'	
Lamin A	Forward<br>Reverse	5'-TCTTCTGCCTCCAGTGTCACG-3'<br>5'-AGTTCTGGGGGCTCTGGGT-3'	
GAPDH	Forward<br>Reverse	5'-GAGCGAGATCCCTCCAAAAT-3'<br>5'-CATCACGCCACAGTTTCC-3'	
PPAR-γ	Forward<br>Reverse	5'- GTCACACTCTGACAGGAGCC-3’<br>5'- CACCGCTTCTTTCAAATCTTGT-3’	
C/EBPα	Forward<br>Reverse	5'-CTAACTCCCCCATGGAGTCGG-3'<br>5'-TCTATAGACGTCTCGTGCTCGC-3'	
CD36	Forward<br>Reverse	5'-GCGACATGATTAATGGCACAG-3'<br>5'-GATCCGAACACAGCGTAGATAG-3'	
LPL	Forward<br>Reverse	5′-TAGTTCCAGCAGCAAAGCAGA-3<br>5'- CTGAGAAATCTCTTCCCGCGTC-3′	

Tissue sections immunostaining
4µm tissue sections were deparaffinized and subjected to heat-induced epitope retrieval. For immunohistochemistry, endogenous peroxidase activity was blocked using a solution of 2.5% hydrogen peroxide in methanol, followed by specimen blocking with 1.5% normalized goat serum. Primary antibodies were applied to sections followed by overnight incubation at 4°C. Sections were incubated with secondary antibodies, followed by the label antibody (ABC Elite, Vector Laboratories). Enzymatic activity was revealed using DAB chromogen (Dako Cytomation). Tissue sections were counterstained with Mayers haemotoxylin (Histolab) then mounted with mounting medium for light microscopy (Pertex, Histolab). For immunofluorescence analysis, blocking was performed with 20% normalized goat or donkey serum and/or mouse-to-mouse blocking reagent (Scytek). Primary antibodies were incubated overnight at 4°C. Secondary antibodies were added and sections were counterstained with DAPI or DRAQ5™ (1:1000, ThermoFisher Scientific) prior to mounting (ProLong Gold antifade reagent, Molecular Probes, P36930).
For progerin staining on human sWAT (n = 5, age range: 31-65 years), over 1500 cells were analyzed for positive staining.

Cells immunostaining
Cells were grown on coverslips. After fixation cells were permeabilized in PBS containing 1% NP-40 (ThermoFisher) and blocked in 1% BSA and 0.2% Triton X-100 (Sigma) before incubation with primary antibodies anti-Ki67 and anti-lamin A/C (Mab3211). Since both antibodies were produced in the same host, a sequential staining was performed. First, anti-Ki67 was incubated at R.T for 1 hour, followed by secondary antibody incubation for 40 min at R.T. After extensive washing, anti-lamin A/C was incubated overnight at 4°C and secondary antibody was incubated the next morning in same conditions as above. Cells were counterstained using DAPI before mounting with Prolong Gold (ThermoFisher).

Whole-mount immunostaining
Fresh sWAT from 60-week-old RPE and control animals (males, n = 3/group) were thinly sliced. Cells were permeabilized and non-specific bindings were blocked using 1% BSA, 5%NGS, 0.3% Tween in PBS (B1 Buffer). Samples were first incubated with the primary antibodies, then with the appropriate secondary antibodies, Bodipy® 493/503 (1:5000, ThermoFisher Scientific) and DAPI (1:2000) in B1 Buffer. The whole experiment was conducted at 4°C and between-step-washes were performed in 0.1% Tween in PBS. Specimens were mounted in 80% glycerol in PBS prior to analysis.

Scanning Electron Microscopy and Transmission Electron Microscopy
sWAT from 90-week RPE and wild-type mice (n = 3/group) were dissected and small pieces were fixed in a mix of 2,5% glutaraldehyde + 1% paraformaldehyde in 0.1M phosphate buffer, pH 7.4. Specimens were rinsed in 0,2 M imidazole buffer, pH 7.4 and post-fixed in 2% osmium tetroxide in 0,2M imidazole buffer, pH 7.4. Tissues were briefly rinsed in distilled water and placed in 70% ethanol, then in 95% ethanol. Samples were then dried using carbon dioxide in a critical point dryer (Balzer, CPD 010, Lichtenstein). After drying, specimens were mounted on an aluminium stub and coated with Platinum (Bal-Tec SCD 005).

Transmission Electron Microscopy (TEM)
sWAT from 90-week RPE and wild-type mice (n = 3/group) were dissected and small pieces were fixed in a mix of 2,5% glutaraldehyde + 1% paraformaldehyde in 0.1M phosphate buffer, pH 7.4. Specimens were rinsed in 0,2M imidazole buffer, pH 7.4 and post-fixed in 2% osmium tetroxide in 0,2M imidazole buffer, pH 7.4, dehydrated in ethanol and embedded in LX-112 (Ladd). Semi-thin sections were cut and stained with toluidine blue O and used for light microscopic analysis. Ultra-thin sections (approximately 50-60 nm) were cut by a Leica EM UC 6 (Leica, Austria) and contrasted with uranyl acetate followed by lead citrate.

Image acquisition
Immunohistochemistry imaging was achieved using the Zeiss Axioplan 2 microscope (Carl Zeiss AG, Germany) coupled to the Zeiss Axiocam MRm camera. Images were analyzed with the Image-Pro Insight 9.1 software. Immunofluorescence imaging was done using Nikon A1R and A1+ imaging systems (Nikon Corporation, Japan). Images were analyzed using NIS elements (Nikon Corporation, Japan). For scanning electron microscopy, specimens were analyzed in an Ultra 55 field emission scanning electron microscope (Zeiss, Oberkochen, Germany) at 3 kV. For transmission electron microscopy, samples were examined in a Tecnai 12 Spirit Bio TWIN transmission electron microscope (FEI company, The Netherlands) at 100 kV. Digital images were taken using a Veleta camera (Olympus Soft Imaging Solutions, GmbH, Germany).

Antibodies used for the study

Antibodies	Vendors	Concentrations
Primary antibodies		
Progerin<br>Mouse anti-human (13A4)	Enzo Life Sciences	1:300
Mab3211<br>Mouse anti-human lamin A+C	Millipore	1:50
γH2AX<br>Rabbit anti-phospho histone H2A.X (Ser 139), clone EP854(2)4		1:1500
NeuN<br>Rabbit anti-NeuN		1:75
GFAP<br>Mouse anti-glial fibrillary acidic protein, clone GAS		1:30000
F4/80<br>Rat anti-mouse F4/80, clone CI:A3-1	AbD Serotec	1:50
cC3<br>Rabbit anti-cleaved caspase 3	Cell Signalling	1:200
Ki67<br>Mouse anti-human Ki67, clone MM1	Vector Laboratories	1:150
CD11b<br>Rat APC-conjugated anti-CD11b, clone M1/70	BD Biosciences	0.2µg/10^6 cells
CD31<br>Rat PE-conjugated anti-CD31, clone MEC 13.3		0.2µg/10^6 cells
CD45<br>Rat V500-conjugated anti-CD45		0.2µg/10^6 cells
Secondary antibodies		
Biotinylated-goat anti-rabbit	Invitrogen	Assay dependent concentrations
Biotinylated-goat anti-mouse		
Goat anti-mouse Alexa Fluor 488/555/647	Molecular probes	
Goat anti-rabbit Alexa Fluor 488		

BaseScope

Progerin transcripts were detected using BaseScope (Advanced Cell Diagnostic) in accordance with the manufacturer's protocol. The assay was performed on 30 weeks RPE sWAT (n = 3). Assay specificity was evaluated using a wild-type sWAT section (n = 1), where progerin transcripts were not detected, and a skin section from a transgenic mouse expressing progerin under the control of the Keratin-5 promoter[4] where cytoplasmic perinuclear positive staining was observed. The protocol was adapted as follow: target retrieval was performed for 15 minutes, Protease IV was applied on sections for 30 minutes and Amp 5 was incubated for 45 minutes.

Fluorescence-activated cell sorting analysis (FACS)

Stromal vascular cells (SVCs) extraction for FACS analysis was done on 30-week RPE animals (n = 3) as described in Cho *et al.,* 2014[5]. In brief, sWAT depots were isolated and lymph nodes removed. Tissues were washed in PBS containing 1% anti-anti, then minced into small pieces. Specimens were digested at 37°C for 30 minutes, in a digestion buffer composed of Hank's balanced salt solution with Ca^{2+} and Mg^{2+}, 0.5% bovine serum albumin and 1mg/mL collagenase A. The SVCs obtained were fully dissociated by addition of EDTA to a final concentration of 10mM for 5 minutes. After filtration and centrifugation, adipocytes form a white layer on top while SVCs form a red/white pellet at the bottom. Thus, adipocytes and the supernatant are removed and the SVCs pellet is resuspended in 0.5mL red blood cell lysis buffer for 5 minutes. After centrifugation, the SVCs pellet is resuspended in PBS containing 1mM EDTA, 25mM HEPES and 1% heat inactivated FBS (FACS buffer), and filtered. SVCs are stained with an antibody cocktail containing 0.2µg of each antibody per 10^6 cells in FACS buffer, rinsed and centrifugated twice and finally resuspended in FACS buffer for FACS analysis.

SUPPLEMETARY REFERENCES

1. Chang, J. *et al.* Clearance of senescent cells by ABT263 rejuvenates aged hematopoietic stem cells in mice. *Nat. Med.* **22**, 78-83 (2016).
2. Schmittgen, T.D. & Livak, K.J. Analyzing real-time PCR data by the comparative C(T) method. *Nat. Protoc.* **3,** 1101-1108; 10.1038/nprot.2008.73 (2008).
3. Strandgren, C. *et al.* Transgene silencing of the Hutchinson-Gilford progeria syndrome mutation results in a reversible bone phenotype, whereas resveratrol treatment does not show overall beneficial effects. *FASEB J.* **29,** 3193-3205 (2015).
4. McKenna, T., Rosengardten, Y., Viceconte, N., Baek, J.H., Grochová, D., Eriksson, M. Embryonic expression of the common progeroid lamin A splice mutation arrests postnatal skin development. *Aging Cell.* **13**, 292-302 (2014).
5. Cho, K.W., Morris, D.L. & Lumeng, C.N. Flow cytometry analyses of adipose tissue macrophages. *Methods Enzymol.* **537,** 297-314 (2014).
